# Impact of Three Nonsurgical, Full-Mouth Periodontal Treatments on Total Bacterial Load and Selected Pathobionts

**DOI:** 10.3390/antibiotics11050686

**Published:** 2022-05-19

**Authors:** Mohamed M. H. Abdelbary, Florian Schittenhelm, Sareh Said Yekta-Michael, Stefan Reichert, Susanne Schulz, Adrian Kasaj, Andreas Braun, Georg Conrads, Jamal M. Stein

**Affiliations:** 1 Division of Oral Microbiology and Immunology, Department of Operative Dentistry, Periodontology and Preventive Dentistry, Rheinisch-Westfälische Technische Hochschule (RWTH) Aachen University Hospital, 52074 Aachen, Germany; gconrads@ukaachen.de; 2 Department of Operative Dentistry, Periodontology and Preventive Dentistry, Rheinisch-Westfälische Technische Hochschule (RWTH) Aachen University Hospital, 52074 Aachen, Germany; f.schittenhelm@gmx.de (F.S.); anbraun@ukaachen.de (A.B.); jstein@ukaachen.de (J.M.S.); 3 Private Practice, 52062 Aachen, Germany; 4 Department of Orthodontics, Rheinisch-Westfälische Technische Hochschule (RWTH) Aachen University Hospital, 52074 Aachen, Germany; samichael@ukaachen.de; 5 Department of Operative Dentistry and Periodontology, Martin Luther University, 06108 Halle (Saale), Germany; stefan.reichert@uk-halle.de (S.R.); susanne.schulz@medizin.uni-halle.de (S.S.); 6 Department of Periodontology and Operative Dentistry, University Medical Center, 55131 Mainz, Germany; adrian.kasaj@unimedizin-mainz.de

**Keywords:** periodontal therapy, full-mouth debridement, antiseptics, periodontal pathobionts, *Aggregatibacter actinomycetemcomitans*, *Porphyromonas gingivalis*, *Prevotella intermedia*, *Tannerella forsythia*, total bacterial load

## Abstract

For the treatment of periodontitis stage III/IV, a quadrant/week-wise debridement (Q-SRP) was compared with three full-mouth approaches: full-mouth scaling (FMS, accelerated Q-SRP within 24 h), full-mouth scaling with chlorhexidine-based disinfection (FMD), and FMD with adjuvant erythritol air polishing (FMDAP). The objective of this prospective, randomized study (a substudy of ClinicalTrials.gov, identifier: NCT03509233) was to compare the clinical and microbiological effects of the treatments. In total, 105 patients were randomized to one of the four aforementioned treatment groups, with *n* = 25, 28, 27, and 25 patients allocated to each group, respectively. At baseline and 3 and 6 months after treatment, the clinical parameters, including the pocket probing depths, clinical attachment level, and bleeding on probing, were recorded, and the prevalence of the total bacteria and four periodontal pathobionts (*Aggregatibacter actinomycetemcomitans*, *Porphyromonas gingivalis*, *Prevotella intermedia*, and *Tannerella forsythia*) was determined using real-time quantitative PCR. Concerning the clinical outcomes, all the treatment modalities were effective, but the full-mouth approaches, especially FMDAP, were slightly superior to Q-SRP. Using the FMD approach, the reduction in the bacterial load and the number of pathobionts was significantly greater than for FMS, followed by Q-SRP. FMDAP was the least effective protocol for microbial reduction. However, after a temporary increase 3 months after therapy using FMDAP, a significant decrease in the key pathogen, *P. gingivalis*, was observed. These findings were not consistent with the clinical results from the FMDAP group. In conclusion, the dynamics of bacterial colonization do not necessarily correlate with clinical outcomes after full-mouth treatments for periodontitis stage III/IV.

## 1. Introduction

Periodontitis is a chronic inflammatory condition in which tissue damage results from dysregulated and prolonged inflammatory responses to a persisting subgingival biofilm [1,2]. Without treatment, the tooth-supporting alveolar bone is destroyed, ultimately leading to tooth loss. Several factors can directly (immunocompetence, nutrients, oral hygiene, smoking, and systemic diseases, such as diabetes) and indirectly (age, genetics, socioeconomic status, and iatrogenic factors) influence the progression and the clinical phenotypes of periodontitis. Every individual has a combination of risk factors, including the individual microbiome and inflammasome, that impact pathogen and periodontal soft- and hard-tissue crosstalk [3,4,5]. The so-called red complex of subgingival bacterial species, which includes *Porphyromonas gingivalis* (*P. gingivalis*), *Treponema denticola*, and *Tannerella forsythia* (*T. forsythia*), encompasses the most important pathogens in periodontitis, called periodontal pathobionts [6,7]. Secondarily, the orange complex, comprising species such as *Prevotella intermedia* (*P. intermedia*), *Fusobacterium nucleatum* (*F. nucleatum*), and *Parvimonas micra,* is associated with disease initiation. Adapted to life in deep pockets, the species of the red and orange complexes are usually anaerobic and proteolytic. In addition to these two complexes, the facultatively anaerobic, leukotoxin-producing *Aggregatibacter actinomycetemcomitans* (*A. actinomycetemcomitans*) plays an etiological role in the development of aggressive periodontitis, but its prevalence in the disease is much lower than the aforementioned pathobionts. Notably, these periodontal pathobionts are never found alone or to act independently, but instead are part of a multispecies consortium that triggers the host inflammatory reaction [8].

Finding an adequate therapy for progressive stage III and IV periodontitis is a very high social priority [9]. Subgingival mechanical debridement (scaling and root planing, SRP) is a well-established, effective treatment method. In addition to classic quadrant-wise SRP (Q-SRP), advanced mechanical treatment options have been evaluated to minimize the risk of periodontal pockets being recolonized from other oral niches, such as the mucosal membranes and tongue. For instance, full-mouth scaling (FMS), which consists in an accelerated SRP of all quadrants within 24 h, was developed. Furthermore, a full-mouth disinfection (FMD) approach was introduced, combining FMS with adjunctive antimicrobial chlorhexidine treatment, to reach all the pathogen niches [10]. However, in a Cochrane Review comparing the clinical outcome of FMS and FMD in contrast to Q-SRP, no significant clinical benefit could be determined [11]. In the recent study by Stein et al., on which the present substudy is based, a new treatment strategy was introduced, combining full-mouth disinfection with additional air-polishing (FMDAP) [12]. The authors demonstrated that FMDAP indeed enhanced clinical outcomes compared to conventional Q-SRP in patients with stage III and IV periodontitis. Furthermore, all groups (Q-SRP, FMS, FMD, and FMDAP) showed significant (*p* < 0.05) improvements in all clinical parameters over 3 and 6 months. Interestingly, FMDAP significantly reduced the mean periodontal pocket depth (PPD) in teeth with moderate (PPD 4–6 mm) and deep (PPD > 6 mm) pockets, and also significantly increased the proportions of pocket closure (the proportion of sites changed from PPD > 4 mm to residual PPD ≤ 4 mm without BOP) compared to Q-SRP. The patients treated with FMD showed a significantly higher PPD reduction in deep pockets and a higher percentage of pocket closure after 3 months, but not after 6 months, compared to Q-SRP. Changes in other clinical parameters, such as clinical attachment level (CAL) and bleeding on probing (BOP), did not significantly differ among all the groups. The treatment efficiency (the time and effort to gain one closed pocket) was significantly higher for all the full-mouth protocols (FMDAP, FMD, FMS) than for Q-SRP (*p* < 0.05) [12]. In a related study, investigating the microbiome of a sub-sample of the original study by next-generation sequencing (NGS), the alpha and beta diversities, as well as a few outcome-associated bacterial taxa of the subgingival microbiome, were revealed [13]. In this study, the impact of the nonsurgical treatments Q-SRP, FMD, FMS, and FMDAP on microbial reduction was determined by precisely quantifying the total bacterial load and the number of cells of selected periodontal pathobionts (*P. gingivalis*, *T. forsythia*, *P. intermedia*, and *A. actinomycetemcomitans*). The null hypothesis stated that there were no statistically significant differences among the four treatment approaches in terms of microbial reduction.

## 2. Results

### 2.1. Clinical Benefit of the Three Full-Mouth Debridement Concepts in Comparison to Q-SRP

The clinical results in these patients have already been published [12]; however, they are briefly summarized here to ease discussion later. Notably, the bacterial load and periodontal pathobionts were analyzed in 105 patient samples (for corresponding subgroup data, see Table 1), which made up 61% of the 172 patients who were analyzed for clinical outcomes. However, the clinical outcomes of this subgroup were in agreement with those of the master group (Table 2).

### 2.2. Reduction in Total Bacterial Load and Periodontal Pathobionts

**Figure 1:**
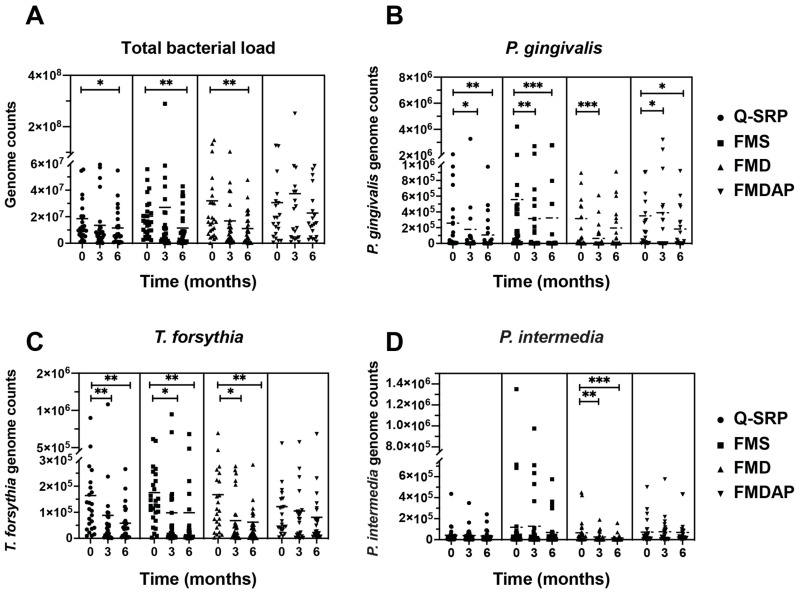
Bacterial load of (**A**) total bacteria, (**B**) *P. gingivalis*, (**C**) *T. forsythia*, and (**D**) *P. intermedia* that were detected at baseline (t0), three (t3), and six (t6) months after one of the following periodontal treatments: Q-SRP, FMS, FMD, and FMDAP. *, **, and *** represent *p* < 0.05, *p* < 0.01, and *p* < 0.001, respectively.

Significant reductions in the total bacterial load were observed between baseline and 6 months after therapy in the three treatment groups Q-SRP (*p* < 0.05), FMS (*p* < 0.01), and FMD (*p* < 0.01) (Figure 1A). Thus, the changes were significantly greater in the FMS and FMD groups than in the Q-SRP. In the FMDAP group, only a trend for total bacterial reduction was observed after 6 months. Interestingly, in the FMS and FMDAP groups, a minor and nonsignificant elevation in bacterial load was detected after 3 months, later decreasing strongly for FMS and, to a minor degree, for FMDAP, 6 months after therapy. Furthermore, all the treatment approaches significantly reduced the *P. gingivalis* load after 3 months (Q-SRP *p* < 0.05, FMS *p* < 0.01, FMD *p* < 0.001, Figure 1B), with the exception of FMDAP, where *P. gingivalis* increased significantly (*p* < 0.05). However, after 6 months, the dynamic changed, and the pathogen numbers decreased compared to baseline for the Q-SRP (*p* < 0.01), FMS (*p* < 0.001), and FMDAP (*p* < 0.05), with the exception of FMD (n.s.). For *T. forsythia*, significant reductions were observed in the three treatment groups, Q-SRP, FMS, and FMD, at 3 months (*p* < 0.01, *p* < 0.05, and *p* < 0.05; respectively) and 6 months (*p* < 0.01, *p* < 0.01, and *p* < 0.01; respectively) after treatment, compared with baseline (Figure 1C). By contrast, the FMDAP group showed only a marginal reduction at 6 months compared to baseline; the latter was not as high as the baseline numbers in other groups, which might be important for interpretation. The orange complex species, *P. intermedia*, was significantly reduced (at 3 months with *p* < 0.01 and 6 months with *p* < 0.001) after applying the FMD treatment strategy (Figure 1D) and only non-significantly when applying the other protocols.

In all four treatment groups, *A. actinomycetemcomitans* was only detected in a few samples at each time point (Supplementary Table S1). Specifically, *A. actinomycetemcomitans* was detected in five (t0), three (t3), and two (t6) samples from the Q-SRP group; in three (t0), three (t3), and two (t6) samples from the FMS group; in four (t0), two (t3), and two (t6) samples from the FMD group; and, finally, in two (t0), one (t3), and two (t6) samples from the FMDAP group. Only five patients (Ac01, Ha11, Ha13, Ha26, and Ha33) were positive for *A. actinomycetemcomitans* at t0, t3, and t6. Of these, three (Ha11: FMS-treated, Ha13: Q-SRP-treated, and Ha26: FMDAP-treated) showed a reduction, one (Ac01: FMD-treated) showed a steady state, and one (Ha33: FMD-treated) even showed an increase in *A. actinomycetemcomitans* cell numbers. However, in two other FMD cases (Ac20 and Ma04), *A. actinomycetemcomitans* was found at baseline and then disappeared throughout t3–t6. It must be emphasized that this low number of *A. actinomycetemcomitans*-positive cases hampered the statistical analysis.

### 2.3. Correlation between Microbiological and Clinical Outcomes

**Figure 2:**
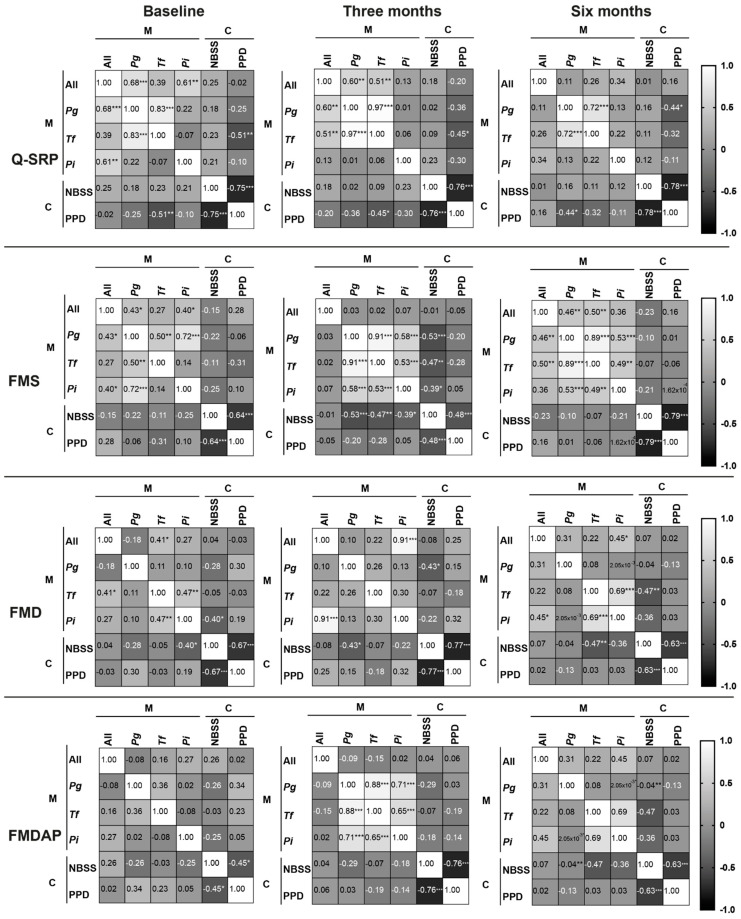
Correlation matrices of the microbiological (M) and clinical (C) outcomes of the four different periodontal treatments: Q-SRP, FMS, FMD, and FMDAP. The bacterial load of total bacteria (All), *P. gingivalis* (*Pg*), *T. forsythia* (*Tf*), and *P. intermedia* (*Pi*) represented the microbiological parameters, while nonbleeding shallow sites (NBSS) and pocket depth (PPD) represented the clinical parameters measured at baseline and at 3 and 6 months for each treatment. *, **, and *** represent *p* < 0.05, *p* < 0.01, and *p* < 0.001, respectively.

The correlation matrices of the microbiological (M) and clinical (C) outcomes of the four different periodontal treatments are presented in Figure 2. Notably, a negative correlation with bacteria (less bacteria, higher % NBSS) for the proportion of nonbleeding shallow sites (NBSS) and a positive correlation with bacteria (less bacteria, less PPD) for the mean periodontal probing depths (PPD) are desirable as treatment outcomes. The results revealed that only slight correlations (near 0) between the microbiological and clinical parameters were detected at baseline in all the treatment groups; only *T. forsythia* versus PPD and *P. intermedia* versus NBSS showed a significant negative correlation in the Q-SRP and FMD group, respectively. Interestingly, only the patients treated with the FMS approach revealed a significant negative correlation among the three key pathogens, *P. gingivalis, T. forsythia*, and *P. intermedia,* and the NBSS detected after 3 months. For the FMD group, only NBSS showed a significant negative correlation with *P. gingivalis* after 3 months. After 6 months, NBSS showed a significant, negative correlation with *T. forsythia* and *P. gingivalis* in the FMD and FMDAP groups, respectively. For PPD, a significant negative correlation with NBSS was detected at all time points for all four treatment groups. PPD showed a statistically significant negative correlation with *T. forsythia* for the Q-SRP group at baseline and 3 months, while at 6 months, this significance vanished and a significant, negative correlation with *P. gingivalis* was detected.

## 3. Discussion

To the best of our knowledge, and according to the results of an advanced PubMed search ((full-mouth OR nonsurgical) AND periodontitis AND PCR, real-time [MeSH Terms]), our study is the first to monitor the number of total bacteria and periodontal pathobionts using state-of-the-art RT-qPCR during nonsurgical, full-mouth periodontal treatments under highly specific conditions. In a separate study by our group, the subgingival microbiome was investigated in a sub-group (*n* = 40) of our patients and by NGS [13]. For the further discussion of the microbiological results, we did not include studies that investigated bacterial profiles by culture (as in Quirynen et al. [14]) or that presented prevalence data only (such as Apatzidou et al. [15]). Instead, we considered studies that investigated changes in microbial abundance and activity after any form of periodontal mechanical treatment as long as appropriate state-of-the-art measurements were performed.

As mentioned in the Results section, according to the clinical outcomes of all the treatment groups (Q-SRP, FMS, FMD, and FMDAP), all the treatments were effective, with no significant differences between the full-mouth approaches. However, FMDAP improved the clinical outcomes more than Q-SRP for moderate and deep pockets after 6 months [12]. In this study, we investigated whether these clinical improvements were supported by microbiological data in terms of total bacterial load and/or the abundance of selected periodontal pathobionts (*P. gingivalis*, *P. intermedia*, *T. forsythia*, and *A. actinomycetemcomitans*). Our findings suggest that the clinical improvement in all the groups can partially be explained by the reduction in total bacterial load and three out of four pathobionts 3 and 6 months after therapy. Considering *A. actinomycetemcomitans*, the number of positive samples was too low to draw any conclusions. As speculated by Stein et al. [12], the mechanical removal of the total bacteria and biofilm mass could have reduced the inflammatory reaction, reducing, in turn, the vasodilatation and BOP in all the treatment groups. In addition, a reduced treatment time and higher efficiency were found for FMS, but no further clinical improvement could be achieved compared to Q-SRP. Notably, an increase in the total bacterial load and in *P. intermedia* was detected after 3 months for the FMS therapy, and a significant decrease was measured after 6 months. Such an increase before a reduction has not been demonstrated for nonsurgical periodontal treatments before, except by two other comparable studies that applied the RT-qPCR approach [16,17]. Rodrigues et al. collected the clinical and microbiological data (at baseline, 45 days after nonsurgical (Q-SRP-like), and both before and after one month of antibiotic treatments) of 15 patients suffering from aggressive periodontitis. The authors showed that the numbers of red-complex bacteria was reduced, while the mean numbers of *A. actinomycetemcomitans* and *Dialister pneumosintes* increased by a factor of 17 (from 10^3^ to 1840 × 10^4^) and 23 (0.47 to 10.9 × 10^4^), respectively [16]. Furthermore, Spooner et al. investigated the load and anabolic activity (deduced from rRNA/genomic DNA ratios) of *P. gingivalis* and *Filifactor alocis* after Q-SRP-like mechanical treatment [17], applying RT-qPCR. They demonstrated a decrease in bacterial load and activities after treatment; however, in 8–40% of the individual samples, both markers increased after the mechanical stimulus. Taken together, the data suggest that the composition of bacterial species and the number of bacterial pathogen cells, including periodontal pathobionts, might be reconstituted and increase, respectively, after intense hygiene and mechanical treatment procedures. This might be due to the intense mechanical stimulation during both Q-SRP and FMS therapies, which might temporarily increase local inflammation and sulcus fluid, which acts as a nutritional source of pathobionts. A previous study, by Graziani et al., in 2015, revealed that FMS therapy induced a greater acute inflammatory response than Q-SRP therapy [18].

Thone-Muhling et al. quantified the peri-implantitis-associated total bacterial load and the number of selected pathogens after 24 h and 2, 4, and 8 months of full-mouth scaling without (corresponding to FMS) and with (FMD) chlorhexidine. No correlations between clinical and microbiological data were found, and the bacterial reduction was only temporary, with a tendency towards recolonization during the study period. Notably, the microbial biofilm became re-established rapidly, starting at 24 h after treatment, even significantly exceeding the baseline value after 8 months in the FMS, but not in the FMD group [19]. In this study, FMD was performed according to the protocol described by Quirynen et al. (1998), which included the repeated use of 0.2% chlorhexidine as a rinsing and tonsil spray up to 2 months after treatment and the repeated subgingival application of 1% chlorhexidine gel after full-mouth debridement. Although the overall added value of chlorhexidine remains unclear, an increasing number of studies support its role in decelerating bacterial regrowth and recolonization. Our results revealed that the total bacterial load (at 6 months) and *P. gingivalis* (at 3 months) could be significantly reduced by using the FMD approach, whereas for *T. forsythia* and *P. intermedia*, a significant reduction was recorded at both 3 and 6 months after the FMD treatment. Nevertheless, the overall reduction in *P. gingivalis* by FMS seems to be superior to that achieved with the FMD treatment (*p* < 0.01 at 3 months and *p* < 0.001 at 6 months versus *p* < 0.001 at 3 months only).

The adjunctive effect of chlorhexidine on bacterial profiles in a direct comparison of FMS and FMD using RT-qPCR has not been investigated to date. However, in a previous study, Afacan et al. (2019) reported that the total bacterial count and numbers of *P. gingivalis*, *P. intermedia*, *T. forsythia*, and *F. nucleatum* were significantly (*p* < 0.05) reduced after Q-SRP and FMD, but that FMD treatment provided the lowest numbers for all bacterial measurements and that significantly reduced levels of *A. actinomycetemcomitans* were detected in subgingival plaque samples at 3 and 6 months, pointing to a broad antiseptic effect of chlorhexidine [20].

Finally, while Q-SRP, FMS, and FMD fulfilled the expectations of microbial reduction, the microbial efficacy of FMDAP treatment was not as high, which contrasts with the clinical outcome reported by Stein et al. [12]. A significant increase in *P. gingivalis* (*p* < 0.05) was detected 3 months after FMDAP treatment, which was followed by a significant decrease (*p* < 0.05) at 6 months compared to baseline. The correlation matrix of the microbiological and clinical responses revealed a spectrum from near 0 to negative values, which were significant mainly for NBSS versus *P. gingivalis*, *T. forsythia,* and *P. intermedia* at 3 months after FMS treatment. The missing correlation between the microbiological and clinical parameters could be explained by the fact that periodontal treatments cannot eradicate, but only reduce, pathobionts. Therefore, many organisms are allowed to persist, along with good clinical responses. An alternative explanation could be sampling fluctuation at the infection/probing sites.

This is the first study, to our knowledge, to include such a correlation matrix for the microbiological and clinical outcomes of nonsurgical, full-mouth periodontal treatments. However, using only NBSS and PPD as representative parameters for the clinical outcomes might be considered as a limitation, rendering the evaluation of correlation models challenging. Therefore, future investigations need to include further parameters, such as measuring the expression levels of inflammatory cytokines and correlating them with the clinical and microbiological outcomes of different treatments.

In conclusion, this study demonstrated that three nonsurgical, full-mouth periodontal treatments (Q-SRP, FMS, and FMD) significantly reduced total bacterial load and selected pathobionts. In contrast to the clinical outcome, the reduction in bacterial load and levels of selected pathobionts was restricted by using the FMDAP treatment approach, and the significant reduction in *P. gingivalis* was postponed. According to these findings, the correlation between the microbiological and clinical outcomes of the investigated periodontal treatments is limited.

## 4. Materials and Methods

### 4.1. Study Design

All treatment strategies were applied in a randomized, multicenter, clinical trial (ClinicalTrials.gov: NCT03509233). Ethical approval was obtained from the ethics committee of the University of Aachen, Germany (EK 046/16). Details of the master study have already been published [12]. Here, in a pathogen-directed part of the master study, subgingival samples from a total of 105 patients suffering from periodontitis stage III or IV were included. This was a subgroup of all patients included in the clinical study (190) and of all patients analyzed for clinical improvement (172). The 105 patients here were randomized at three different German university dental departments (Department of Operative Dentistry, Periodontology and Preventive Dentistry, University Hospital (RWTH), Aachen; Department of Periodontology and Operative Dentistry, University Medical Centre, Mainz; and Department of Operative Dentistry and Periodontology, Martin-Luther-University, Halle/Saale) and into four treatment groups: Q-SRP (quadrant-wise subgingival scaling and root planing, SRP, clockwise, in four sessions, with an interval of one week between each quadrant without the use of antiseptics; *n* = 25); FMS (full-mouth SRP within 24 h without the use of antiseptics; *n* = 28); FMD (full-mouth SRP within 24 h with disinfection using antiseptic applications, according to the protocol described by Quirynen et al., 1998 [10]; *n* = 27); and FMDAP (FMD combined with the use of subgingival erythritol air-polishing; *n* = 25). For randomization and allocation concealment, sealed envelopes were used (details described in Stein et al. [12]). All patients in this study received the same oral hygiene instructions and used the same hygiene equipment throughout the study period. At each center, all patients were examined by calibrated examiners who were blinded to the treatment approach. Periodontal examinations included the recording of PI, GI (according to the standard described by Löe 1967) [21], PPD, CAL, and BOP. Furthermore, the proportion of NBSS with PPD ≤ 4 mm without BOP was assessed. The subgingival bacterial biofilm samples were collected from the deepest pocket of each quadrant by the same examiners, applying sterile paper points (Hain Lifescience, Nehren, Germany) that were inserted for 20–30 s. For each patient, all four samples per time point were pooled into a single tube that was stored at −20 °C (short term) or −80 °C (long term), respectively, until DNA isolation and analysis.

### 4.2. Microbiological Analysis

The genomic DNA was extracted from the pooled samples using QIAamp^®^ DNA mini kit (Qiagen, Hilden, Germany) following the manufacturer’s instructions. The microbial diversities among samples were evaluated via a real-time quantitative polymerase chain reaction, RT-qPCR, which was performed using a QuantStudio 3 block cycler in microtiter plates (Thermo Fisher Scientific, Dreieich, Germany). For each sample, we measured the genome number of total bacteria (domain bacteria) and for each of the four periodontal pathogens, *A. actinomycetemcomitans*, *P. gingivalis*, *P. intermedia*, and *T. forsythia*, using primers listed in Table 3. DNA stock suspensions of *Streptococcus oralis* ATCC 35037 (8.0 × 10^8^/mL), *A. actinomycetemcomitans* strain ATCC 33384 (2.25 × 10^8^/mL), *P. gingivalis* ATCC 33277 (2.8 × 10^8^/mL), *P. intermedia* ATCC 25611 (2.98 × 10^8^/mL), and *T. forsythia* ATCC 43037 (5.0 × 10^7^/mL) were serially diluted in tenfold steps with nuclease-free water and served as bacterial reference strains of the standard curve for RT-qPCR. The PowerUp™ SYBR™ Green Master Mix (Thermo Fisher Scientific, Dreieich, Germany) was used to create a reaction mix. Each microtiter plate well contained 20 µL of the reaction mix with the following components: PowerUp™ SYBR™ Green Master Mix (10 µL), forward primer (0.1 µL of 100 µM), reverse primer (0.1 µL of 100 µM), nuclease-free water (8.8 µL), and template (1 µL). The qRT-PCR was performed under the following conditions: initial denaturation at 95 °C (2 min), followed by 40 cycles of 95 °C (10 s), Ta (see Table 1, 15 s), 72 °C (25 s), and a final cycle of 95 °C (10 s), 60 °C (1 min), and 95 °C (15 s) for dissociation curve (melt curve) stage. As a negative control, nuclease-free water, was added instead of the template.

### 4.3. Statistical Analysis

Data were analyzed using GraphPad Prism, version 9.1.2 (San Diego, CA, USA). The data were paired (as they represented patients’ samples over time) and not normally distributed; therefore, the nonparametric ANOVA for repeated-measurement Friedman test was performed. A correlation matrix between the microbiological and selected clinical (proportion of NBSS and mean PPD) outcomes was determined by computing two-tailed Pearson correlations. To evaluate the degree of correlation, a higher correlation coefficient between 0 and 1 suggested a stronger positive correlation, while correlation coefficient values between 0 and −1 suggested a stronger negative correlation. It is important to consider that for NBSS, a negative correlation with bacteria (less bacteria, higher % NBSS) and, for the mean PPD, a positive correlation with bacteria (less bacteria, less PPD) are desirable as treatment outcomes. The statistical significance threshold was set at *p* ≤ 0.05.

## Figures and Tables

**Table 1:** Patient data recorded at the baseline examination.

Variable	Q-SRP (*n* = 25)	FMS (*n* = 28)	FMD (*n* = 27)	FMDAP (*n* = 25)
Age (years)	61.1 ± 9.9	56.4 ± 10.4	59.2 ± 12.4	55.0 ± 11.8
Gender (male/female)	19/6	17/11	15/12	13/12
Smokers (*n*)	6	8	8	3

Q-SRP: quadrant-wise subgingival scaling and root planing (SRP); FMS: full-mouth SRP within 24 h without the use of antiseptics; FMD: FMS with disinfection using antiseptic applications; FMDAP: FMD combined with the use of subgingival erythritol air polishing.

**Table 2:** Full-mouth clinical parameters at baseline and follow-up visits.

Variable	Timepoint	Q-SRP (*n* = 25)	FMS (*n* = 28)	FMD (*n* = 27)	FMDAP (*n* = 25)
PI	Baseline	1.21 ± 0.52	1.17 ± 0.60	1.17 ± 0.54	1.23 ± 0.68
	3 months	0.75 ± 0.43	0.79 ± 0.45	0.50 ± 0.40	0.46 ± 0.35
	6 months	0.84 ± 0.51	0.84 ± 0.52	0.76 ± 0.48	0.43 ± 0.32
GI	Baseline	1.08 ± 0.56	1.09 ± 0.65	1.07 ± 0.54	1.11 ± 0.47
	3 months	0.73 ± 0.54	0.71 ± 0.58	0.51 ± 0.41	0.37 ± 0.36
	6 months	0.78 ± 0.56	0.68 ± 0.47	0.48 ± 0.36	0.34 ± 0.25
PPD (mm)	Baseline	3.75 ± 0.68	3.74 ± 0.54	4.09 ± 0.61	4.09 ± 0.69
	3 months	3.33 ± 0.60	3.32 ± 0.44	3.45 ± 0.57	3.36 ± 0.71
	6 months	3.32 ± 0.55	3.31 ± 0.53	3.34 ± 0.91	3.33 ± 0.73
CAL (mm)	Baseline	4.32 ± 0.85	4.23 ± 0.71	4.92 ± 0.89	4.92 ± 1.01
	3 months	3.91 ± 0.73	3.90 ± 0.64	4.40 ± 0.83	4.37 ± 1.09
	6 months	3.94 ± 0.70	3.92 ± 0.76	4.34 ± 0.91	4.38 ± 1.14
BOP (%)	Baseline	33.33 ± 18.34	34.56 ± 19.10	34.79 ± 15.23	40.60 ± 22.30
	3 months	16.39 ± 15.70	17.37 ± 15.10	12.50 ± 9.22	12.80 ± 11.56
	6 months	19.33 ± 15.71	16.30 ± 14.76	14.78 ± 12.46	11.91 ± 10.43
NBSS (%)	Baseline	57.48 ± 20.64	56.37 ± 19.65	53.20 ± 18.76	49.83 ± 20.54
	3 months	73.80 ± 17.13	74.47 ± 15.63	76.62 ± 14.96	77.37 ± 15.70
	6 months	72.42 ± 16.82	76.19 ± 16.63	75.39 ± 16.18	79.26 ± 14.95

BOP, bleeding on probing; CAL, clinical attachment level; GI, gingival index; PI, plaque index; PPD, probing pocket depths; NBSS, non-bleeding shallow sites (PD ≤ 4 mm without BOP).

**Table 3:** List of primers used in this study.

Target	Primer	Sequence 5′–3′	Amplicon Size (bp)	Ta (°C)	Reference
All bacterial species	Nadkarni F	TCCTACGGGAGGCAGCAGT	466	60	[22]
	Nadkarni R	GGACTACCAGGGTATCTAATCCTGTT			
Forward primer for all following species-specific qPCRs	pF-1	AGAGTTTGATCCTGGCTCAG			[23]
*P. gingivalis*	Pg-R	CAATACTCGTATCGCCCGTTATTC	478	59	[24]
*A. actinomycetemcomitans*	Aa-R	GGCATGCTATTAACACAC	469	54	[25]
*P. intermedia*	Pi-R	GTTGCGTGCACTCAAGTCCGCC	660	56	[26]
*T. forsythia*	Tf-R	TGCTTCAGTGTCAGTTATACCT	478	56	[27]

T_a_ = annealing temperature of the primer pairs.

## Data Availability

The data are presented within the article and Supplementary Material Table S1.

## Supplementary Materials

The following supporting information can be downloaded at: https://www.mdpi.com/article/10.3390/antibiotics11050686/s1. Table S1. Sample-specific detection of *Aggregatibacter actinomycetemcomitans* sorted by treatment.
