# Supplementary material for: Impact of Three Nonsurgical, Full-Mouth Periodontal Treatments on Total Bacterial Load and Selected Pathobionts
Source: Antibiotics (Basel). 2022 May 19;11(5):686. doi: 10.3390/antibiotics11050686 (PMC9138013; doi:10.3390/antibiotics11050686)
Supplement: Supplementary file 1 [file antibiotics-11-00686-s001.zip › antibiotics-1491130-supplementary.pdf]

Supplementary Table S1. Sample-specific detection of *Aggregatibacter actinomycetemcomitans* sorted by treatment.

Treatment	Sample	t0	t3	t6	Treatment	Sample	t0	t3	t6
**Q-SRP**	Ac15	0	0	0	**FMD**	Ac01	782	340	603
**Q-SRP**	Ac16	727	0	170	**FMD**	Ac05	0	0	0
**Q-SRP**	Ac18	0	0	0	**FMD**	Ac10	0	0	0
**Q-SRP**	Ac25	0	0	0	**FMD**	Ac14	0	0	0
**Q-SRP**	Ac32	0	0	0	**FMD**	Ac20	10132	0	0
**Q-SRP**	Ac35	0	1311	0	**FMD**	Ac26	0	0	0
**Q-SRP**	Ac36	0	0	0	**FMD**	Ac30	0	0	0
**Q-SRP**	Ac37	0	0	0	**FMD**	Ac52	0	0	0
**Q-SRP**	Ac40	0	0	0	**FMD**	Ac56	0	0	0
**Q-SRP**	Ac43	0	0	0	**FMD**	Ac64	0	0	0
**Q-SRP**	Ac47	0	0	0	**FMD**	Ac65	0	0	0
**Q-SRP**	Ac49	0	0	0	**FMD**	Ac68	0	0	0
**Q-SRP**	Ac50	202	0	0	**FMD**	Ac76	0	0	0
**Q-SRP**	Ac58	0	0	0	**FMD**	Ac77	0	0	0
**Q-SRP**	Ac59	0	0	0	**FMD**	Ac78	0	0	0
**Q-SRP**	Ac60	0	0	0	**FMD**	Ac79	0	0	0
**Q-SRP**	Ac61	0	0	0	**FMD**	Ac82	0	0	0
**Q-SRP**	Ha13	1666	1254	538	**FMD**	Ha2	0	0	0
**Q-SRP**	Ha27	0	0	0	**FMD**	Ha5	0	0	0
**Q-SRP**	Ha31	0	55	0	**FMD**	Ha8	0	0	0

**Q-SRP**	Ha32	0	0	0	**FMD**	Ha16	0	0	0
**Q-SRP**	Ma02	0	0	0	**FMD**	Ha23	0	0	0
**Q-SRP**	Ma05	0	0	0	**FMD**	Ha33	258	92	8996
**Q-SRP**	Ma07	167	72242	0	**FMD**	Ma01	0	0	0
**Q-SRP**	Ma13	111	0	0	**FMD**	Ma04	1238	0	0
**FMS**	Ac03	0	0	0	**FMD**	Ma19	0	0	0
**FMS**	Ac04	0	0	0	**FMD**	Ma20	0	0	0
**FMS**	Ac08	0	4015	109	**FMDAP**	Ac02	0	0	0
**FMS**	Ac12	0	0	0	**FMDAP**	Ac06	0	0	0
**FMS**	Ac22	21183	0	0	**FMDAP**	Ac07	0	0	0
**FMS**	Ac27	0	0	0	**FMDAP**	Ac09	0	0	0
**FMS**	Ac34	0	0	0	**FMDAP**	Ac17	0	0	0
**FMS**	Ac41	172	116	0	**FMDAP**	Ac19	0	0	0
**FMS**	Ac42	0	0	0	**FMDAP**	Ac24	17910	0	0
**FMS**	Ac44	0	0	0	**FMDAP**	Ac29	0	0	0
**FMS**	Ac45	0	0	0	**FMDAP**	Ac31	0	0	0
**FMS**	Ac54	0	0	0	**FMDAP**	Ac38	0	0	0
**FMS**	Ac55	0	0	0	**FMDAP**	Ac39	0	0	0
**FMS**	Ac67	0	0	0	**FMDAP**	Ac66	0	0	0
**FMS**	Ac69	0	0	0	**FMDAP**	Ac74	0	0	0
**FMS**	Ac70	0	0	0	**FMDAP**	Ac75	0	0	0
**FMS**	Ac71	0	0	0	**FMDAP**	Ac81	0	0	0
**FMS**	Ac72	0	0	0	**FMDAP**	Ha3	0	0	0
**FMS**	Ac73	0	0	0	**FMDAP**	Ha10	0	0	579

**FMS**	Ha4	0	0	0	**FMDAP**	Ha19	0	0	0
**FMS**	Ha11	13492	88	220	**FMDAP**	Ha24	0	0	0
**FMS**	Ha17	0	0	0	**FMDAP**	Ha26	3451	494	62
**FMS**	Ha21	0	0	0	**FMDAP**	Ma03	0	0	0
**FMS**	Ha22	0	0	0	**FMDAP**	Ma12	0	0	0
**FMS**	Ha29	0	0	0	**FMDAP**	Ma14	0	0	0
**FMS**	Ma08	0	0	0	**FMDAP**	Ma15	0	0	0
**FMS**	Ma09	0	0	0	**FMDAP**	Ma18	0	0	0
**FMS**	Ma11	0	0	0					
